# Auxin Controls Root Gravitropic Response by Affecting Starch Granule Accumulation and Cell Wall Modification in Tomato

**DOI:** 10.3390/plants14071020

**Published:** 2025-03-25

**Authors:** Huabin Liu, Yue Wu, Jiahui Cai, Lele Xu, Cheng Zhou, Chengliang Wang

**Affiliations:** 1 College of Life and Health Sciences, Anhui Science and Technology University, Bengbu 233100, China; wy16605662883@126.com (Y.W.); caijh@ahstu.edu.cn (J.C.); 19556909605@163.com (L.X.); zhoucheng@njau.edu.cn (C.Z.); 2 College of Life Sciences, Anhui Normal University, Wuhu 241000, China

**Keywords:** root gravitropism, auxin biosynthesis, auxin transport, statolith, transcriptome, *Solanum lycopersicum*

## Abstract

The gravitropic growth of roots is crucial for plants to adapt to terrestrial environments and acquire nutrients from the soil. Tomatoes are a vital economic crop that requires abundant water and nutrients for growth and development. However, there are few reports on the regulatory mechanisms of tomato root gravitropism, particularly auxin-mediated root gravitropic growth. Here, we revealed the signaling pathway of auxin regulating tomato root gravity response through exogenous auxin and auxin inhibitor treatment combined with transcriptome profiling. Our data underscore the necessity of auxin biosynthesis, transport, and optimal levels for the gravitropic growth of tomato roots. Treatment with exogenous auxin or auxin biosynthesis inhibitors diminished gravitropic response in tomato roots. Conversely, treatment with an auxin transport inhibitor led to a robust agravitropic response. Furthermore, we observed that auxin controls root gravitropic growth by establishing concentration gradients and influences root perception of gravity signals by positively regulating starch granule accumulation. Treatment with the exogenous auxin NAA heightened starch synthesis, while exogenous application of the auxin biosynthesis inhibitor yucasin dampened starch synthesis in tomato roots. Our study observed a slow gravitropic response in cultivated cherry tomato (Aisheng) roots. Time series analysis showed that tomato roots bend toward gravity at a slower rate. Transcriptome analysis revealed that many (2770) differentially expressed genes (DEGs) were identified in roots following 36 h of gravity stimulation. In contrast, only 58 DEGs were detected after 3 h of gravity stimulation, further supporting the slow gravitropic response phenotype of tomato roots. GO and KEGG analysis highlighted auxin response, starch and sugar metabolism, and cell wall modification as the major regulatory pathways involved in the gravitropic response and growth of tomato roots. Our results indicate that auxin mediates root sensing of gravity signals through feedback regulation of starch accumulation and controls root gravitropic bending by regulating the expression of cell wall modification-related genes.

## 1. Introduction

Gravitropism is a pivotal trait enabling plants to sense environmental cues and adapt to diverse conditions. Plant gravitropic growth responses facilitate the strategic placement of root systems in the soil, optimizing water and mineral ion absorption and providing essential nutrients and structural support for aboveground plant parts [1]. Root gravitropism involves sequential stages: gravity signal perception and transduction, establishment of an auxin concentration gradient, and stimulation of root tip bending towards the gravity vector [2,3,4,5]. Starch granules, acting as statoliths, within the columella cells serve as crucial gravity sensors in plants [6,7]. When the root deviates from the gravity vector, statoliths swiftly settle to the bottom of columella cells due to the relatively high proportion of starch granules [8,9].

The gravity signal is perceived by starch granules in root columella cells, while the gravitropic response and bending occur in the elongation zone (EZ) [10]. Studies indicate that auxin is instrumental in mediating root gravitropic response and bending [4]. Therefore, statolith sedimentation is suggested to trigger auxin transport and the establishment of an auxin gradient across the lateral root cap (LRC) [11,12]. Starchless *pgm-1* and *adg1-1* mutants, lacking amyloplasts, struggle to create asymmetric auxin distribution, resulting in defects in root gravitropic response [13,14]. A recent study indicates that amyloplasts induce the relocalization of LAZY and LAZY1-LIKE proteins (LZY) on the plasma membrane through sedimentation [15]. LAZY, in turn, induces asymmetric auxin distribution and differential root growth [7,12,16]. Although the critical roles of auxin in the gravitropic response are recognized, its involvement in starch granule accumulation and gravity perception remains unclear.

The lateral auxin gradient crucial for gravitropic processes is initiated by auxin transport, facilitated by the carriers of AUX1/LAX and PIN proteins [4,17,18]. When vertically growing plant seedlings experience a change in orientation (gravistimulated), PIN3 and PIN7 relocate to the bottom of columella cells, directing auxin flow to the LRC [19,20]. AUX1 and PIN2 are responsible for transporting auxin from LRC to the EZ [4,21,22,23]. The lateral auxin gradient is induced by gravity, inhibiting cell expansion on the lower root sides and prompting root bending [4,24]. Auxin plays an important role in root gravitropism by establishing an asymmetric distribution; however, how auxin contributes to the root gravitropic response and subsequent gravitropic bending growth remains unclear.

Auxin biosynthesis also contributes to root development and gravitational response [25,26]. In Arabidopsis, tryptophan aminotransferase of Arabidopsis (TAA) and YUCCA (YUC) serve as the rate-limiting enzymes in auxin biosynthesis [27]. Initially, tryptophan is catalyzed by the TAA family to form indole-3-pyruvic acid (IPA), and then IPA is further catalyzed by the flavin monooxygenase-like protein YUC family to indole-3-acetic acid (IAA) [27,28]. Mutants such as *taa1* and *taa1 tar2* exhibit defects in both root development and gravitropic response [28,29]. Although the *yuc* single mutant does not display apparent growth or developmental defects, the *yucQ* mutants show deficiencies in root development and gravity response [25]. Moreover, the synthesis of local auxin is essential for root gravitropism, and auxin from the aboveground parts is insufficient to support the root gravitropic response [25]. However, the precise involvement of the auxin biosynthesis pathway in gravitropic perception and the subsequent gravitropic response of roots remains poorly understood.

Despite extensive research on root gravitropism in model plants such as Arabidopsis and rice, there is a paucity of studies focusing on the root gravity response in tomatoes. Unlike Arabidopsis roots, which exhibit a rapid response to gravitropic stimuli, tomato roots demonstrate a delayed reaction. To elucidate the underlying mechanisms governing this phenomenon, we conducted an analysis combining phenotypic observations and transcriptome. This study delved into the roles of auxin in the gravitropism of tomato roots. Alterations in auxin levels (content) induced by the application of exogenous auxins or auxin biosynthesis inhibitors revealed the significance of optimal auxin levels in the gravitropic responses of tomato roots. Disrupting polar auxin transport through auxin transport inhibitor application led to pronounced defects in the gravitropic response of roots. Besides its involvement in gravitropic response and bending growth, auxin governs starch accumulation at the root tip, indicating its pivotal role in gravitropic perception. The combination of the expression of the auxin reporter gene *DR5* and transcriptome analysis can elucidate the involvement of starch and sucrose metabolism, auxin signal, and cell wall modification pathways in the gravitropic response of tomato roots. Our results suggest a regulatory mechanism for the gravitropic response of tomato roots that is mediated by the starch granule–auxin–cell wall modification signaling pathway.

## 2. Results

### 2.1. Lagging Root Gravitropic Response in the Cultivated Cherry Tomatoes (Aisheng)

**Figure 1:**
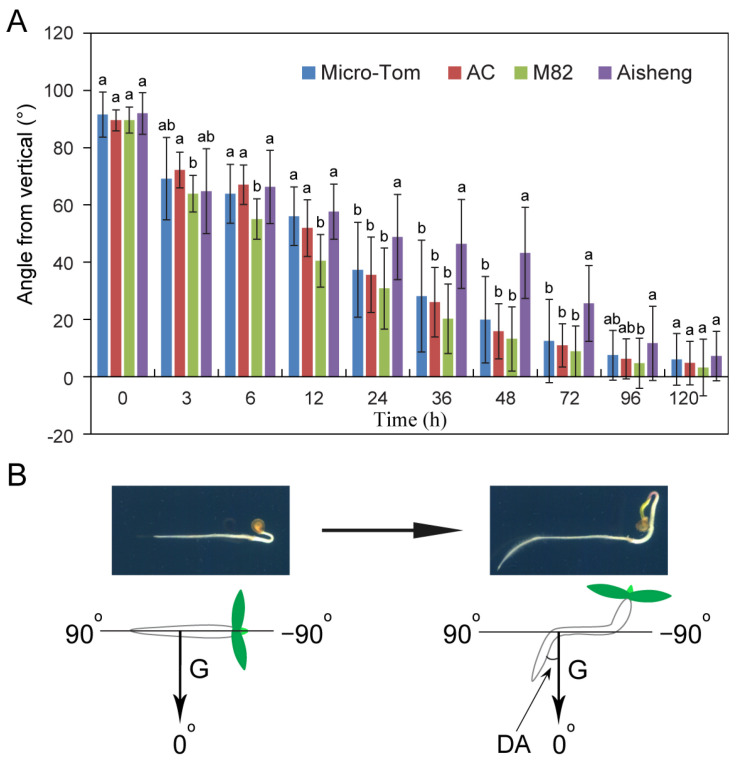
Root gravitropic response of tomato. (**A**) Time series analysis illustrating the gravitropic responses of tomato varieties (Micro-Tom, AC, M82, and Aisheng). Four-day-old tomato seedlings underwent a 90° rotation for gravity stimulation. Statistical analysis of root tip curvature angles, deviating from the gravity vector, was conducted for each time point. Values denote mean ± SD of three biological replicates (8–10 seedlings per replicate). Different letters indicate statistical differences (*p* < 0.05) as determined by Duncan’s multiple-range tests for one-way ANOVA. (**B**) Schematic representation of the gravity stimulation experimental model. Gravity vector is marked by black arrows. Deviation angle (DA) is defined as the angle between the root tip and the gravity vector.

To investigate the characteristics of root gravitropism in tomatoes, we conducted experiments using varieties with diverse backgrounds, including two large modern cultivated types (M82 and AC) and two miniature varieties (Micro-Tom and Aisheng). The results revealed that all four tomato species, including cultivated tomato Micro-Tom, Aisheng, AC, and M82, exhibited responsiveness to gravity stimulation signals (Figure 1). However, distinctions in the gravitropic response among different tomato cultivars were observed. Within the initial 3 h, all four tomato species promptly responded to gravitational stimulation, with the root tips deviating approximately 20° from the horizontal direction (towards the gravity vector) (Figure 1A). Notably, the root tip of M82 and Aisheng tomatoes exhibited a bending of over 25° (Figure 1A). The gravitropic bending rate analysis indicated that Aisheng tomatoes exhibited a significantly higher bending rate during the 0–3 h gravity stimulation than the other two tomato varieties (Micro-Tom and AC; Figure S1). In contrast to Micro-Tom, the gravity-induced gravitropic bending rate of Aisheng tomato roots started to decelerate notably after 12 h of gravity stimulation (Figure S1). By 24 h of gravity stimulation, the gravity-induced bending growth of Aisheng tomato roots lagged behind the other three tomato varieties (Figure 1A). After 72 h of gravity stimulation, the gravity-induced bending growth of Aisheng tomato roots resumed acceleration (Figure S1). By 120 h of gravistimulation, the root bending angles of all four tomato species aligned closely with the gravity vector, completing the response to gravity stimulation (Figure 1). Additionally, M82 exhibited a rapid response to gravity stimulation between 6 and 12 h (Figure 1). In other periods in response to gravity stimulation, no significant differences were detected between Micro-Tom, AC, and M82.

**Figure 2:**
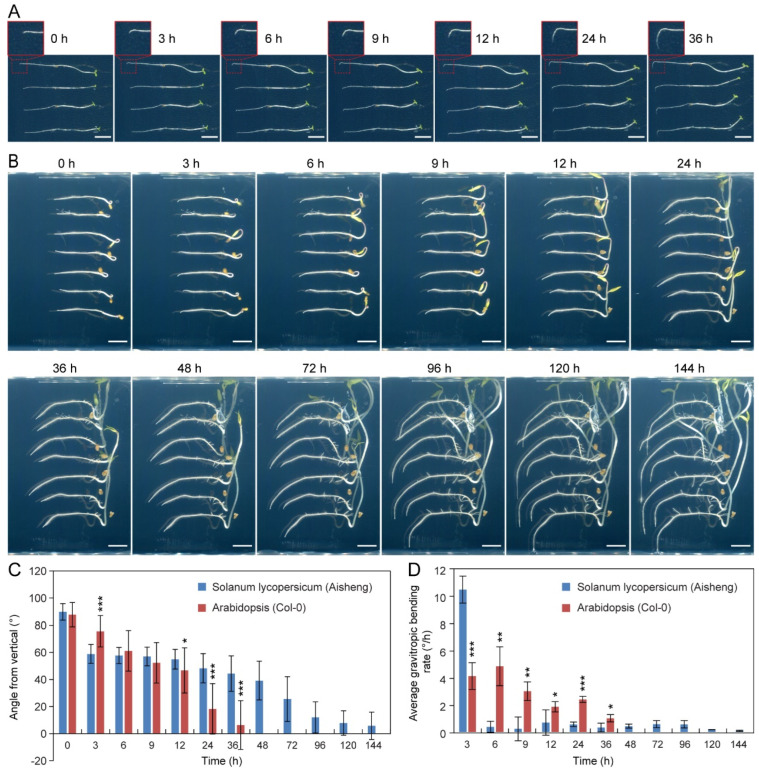
Disparities in gravity response between tomato and Arabidopsis seedlings. (**A**–**D**) Time-series analysis of gravitropic responses in Arabidopsis and tomato roots. Wild-type Arabidopsis (Col-0, (**A**)) and tomato (Aisheng, (**B**)) seedlings at 4 days old underwent a 90° rotation for gravity stimulation. Bars in (**A**) and (**B**) represent 0.5 and 1 cm, respectively. (**C**) Calculations of root bending angles were performed at specified time points. (**D**) Average gravitropic bending rate of tomato and Arabidopsis roots after gravity stimulation. Values represent mean ± SD of three biological replicates (8–10 seedlings per replicate in tomato, 30–35 seedlings per replicate in Arabidopsis). Asterisks denote a significant difference between tomato and Arabidopsis (* *p* < 0.05; ** *p* < 0.01; and *** *p* < 0.001; Student’s *t*-test).

Previous studies have demonstrated the rapid and efficient response of Arabidopsis to gravity signals, accomplishing root bending and growth along the gravity vector in a short duration [30]. To unravel the mechanisms underlying gravitropic responses in two dicotyledonous model plants, the response characteristics of tomato and Arabidopsis to gravistimulation were analyzed comparatively. Time series analysis revealed that Arabidopsis roots maintained a consistently sensitive and rapid growth rate of gravity-induced bending (Figure 2A,C,D). The roots efficiently responded to gravistimulation, aligning with the direction of gravity within 24 h (Figure 2A,C). In contrast, tomato roots exhibited a significantly slower response to gravitational stimulation. The cultivated tomato variety (Aisheng) responded rapidly to the gravitational signal for the initial 3 h and then decelerated (Figure 2B–D). After approximately 120 h, the tomato roots completed the bending towards the gravity vector (Figure 2B). These findings suggest that tomatoes exhibit a swift response to gravitropic stimulation in the early stage but manifest a slower response in the later stage.

### 2.2. Auxin-Driven Regulation of Gravitropic Root Growth in Tomato

**Figure 3:**
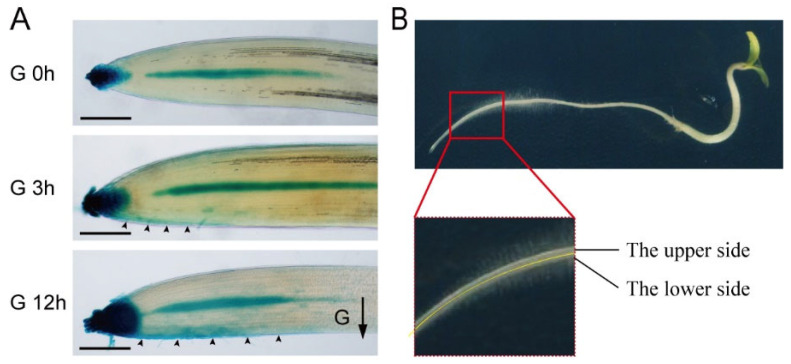
Gravity-induced asymmetric auxin gradients in tomato roots. (**A**) DR5:GUS-expressing tomato seedlings (4 days old) were subjected to a 90° rotation for gravity stimulation for 3 or 12 h. Visualization of DR5:GUS expression in tomato roots. Direction of gravity stimulation indicated by black arrow. (**B**) Schematic depiction of root gravitropic bending and auxin response to gravity stimulation. Bars, 200 μm.

The gravitropic bending growth of roots is orchestrated by asymmetric cell growth in the upper and lower root regions. Central to this process is the asymmetrical auxin accumulation in the EZ, serving as the primary force propelling the curvature growth of the root apex. To elucidate the regulatory mechanism of auxin in root gravitational response in tomatoes, we employed the auxin reporter gene *DR5:GUS* to visualize auxin distribution and accumulation in tomato roots. Following 3 h of gravity stimulation, faint auxin signals were observed in the LRC and meristem on the lower root sides (Figure 3A). Subsequently, after 12 h of gravity stimulation, a substantial auxin accumulation was evident in the lower cells of the LRC, meristematic zone (MZ), and EZ (Figure 3A). This excessive auxin accumulation in the lower cells induced asymmetric growth of the EZ, ultimately steering gravitropic bending (Figure 3). Interestingly, the establishment of asymmetric auxin distribution takes longer in tomatoes compared to our previous reports in Arabidopsis in response to gravitational stimulation [31]. This delay also explains why tomato roots show a slow gravitropic response. These findings suggest that the transport of auxin was caused by gravity stimulation from the root tip to the meristem and EZ was a driving force for root growth bending toward gravity.

### 2.3. Auxin Biosynthesis and Transport Are Required for Root Gravitropic Growth

**Figure 4:**
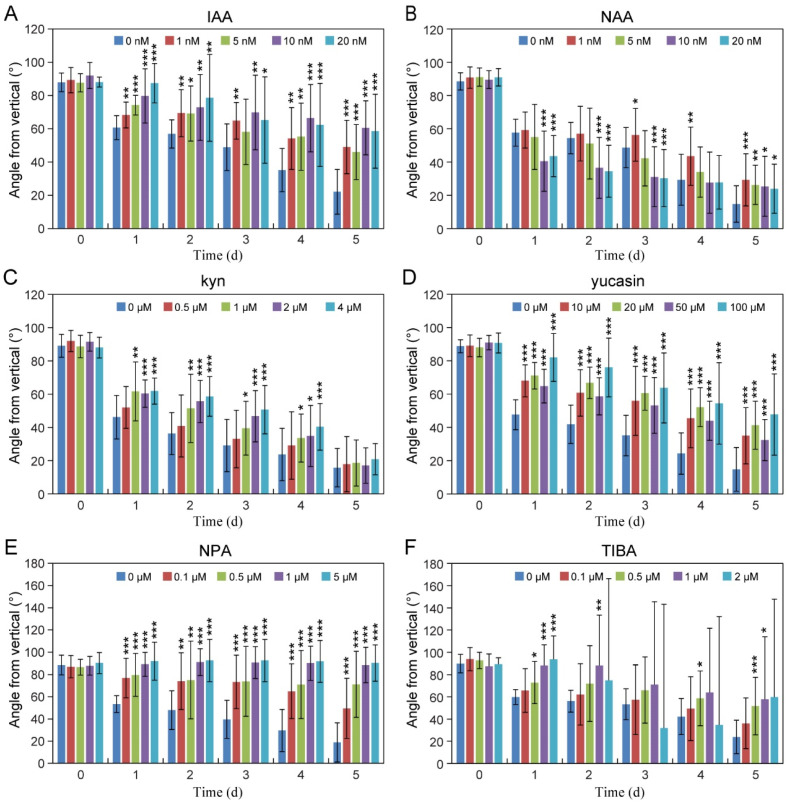
Effect of auxins and auxin biosynthesis and transport inhibitors on root gravitropism. (**A**–**F**) Tomato (Aisheng) seedlings were placed on 1/2MS with varying concentrations of IAA (**A**), auxin analog NAA (**B**), auxin biosynthesis inhibitors kyn (**C**) and yucasin (**D**), or auxin transport inhibitors NPA (**E**) and TIBA (**F**). Seedlings were cultured vertically for 12 h, rotated 90° for gravity stimulation, and root curvature angles were measured. Values denote mean ± SD of three biological replicates (8–10 seedlings per replicate). Asterisks indicate the statistical significance of comparisons with untreated control, * *p* < 0.05; ** *p* < 0.01; and *** *p* < 0.001; Student’s *t*-test.

To explore how auxins regulate the gravitropic bending growth of tomato roots, we analyzed the gravitropic response of roots through auxin biosynthesis, transport, and signaling pathways. Compared to the controls, roots treated with exogenous IAA exhibited a markedly diminished gravitropic response, remaining significantly deviated from the gravity vector after five days of stimulation (Figure 4A). Conversely, treatment with the auxin analog NAA, particularly at higher concentrations (10 and 20 nM), demonstrated a distinct promotion of root gravitropic response during the initial 3 days of gravistimulation (Figure 4B), underscoring the differential functions of distinct auxins in regulating root gravitropism.

Competitive inhibitors of TAA and YUC enzymes, kyn and yucasin, respectively, disrupt the IPA pathway-mediated auxin biosynthesis [32,33]. Applying kyn diminished the roots’ gravitropic response in the initial 3 days (Figure 4C). However, the rate of root bending toward gravity accelerated after four days of gravity stimulation and almost reached the same bending angle toward gravity as that of the controls on the fifth day (Figure 4C). In contrast, yucasin treatment significantly reduced root gravitropic responses (Figure 4D), emphasizing the pivotal roles of the auxin biosynthesis pathway in tomato root gravitropism.

Auxin transport inhibitors, both NPA and TIBA, were employed to evaluate the contribution of PAT to root gravitropism. Seedlings treated with NPA or TIBA exhibited pronounced defects in root gravitropism compared to the controls (CKs; Figure 4E,F). Notably, TIBA-treated seedlings displayed aberrant gravitropism patterns (Figure 4F). Consistent with the gravitropic defect of roots, NAA, NPA, or yucasin-treated seedlings showed a disruption of the lateral auxin gradient across gravistimulated roots (Figure S2). The results collectively highlight the essential interplay of auxin biosynthesis, transport, and optimal concentration levels in orchestrating the root gravitropic response in tomatoes.

### 2.4. Auxin Positively Regulates Starch Accumulation in Tomato Root Tips

Statolith sedimentation is central to plant gravity perception and intricately linked to triggering the gravitropic response in roots. Starch granules serve as crucial mediators, facilitating the conversion of gravitropic signals into intracellular biochemical cues [8]. This process sets the stage for auxin accumulation in the lower root cells, establishing a concentration gradient [11] that propels the asymmetric auxin distribution, driving differential growth and culminating in root gravitropic bending.

**Figure 5:**
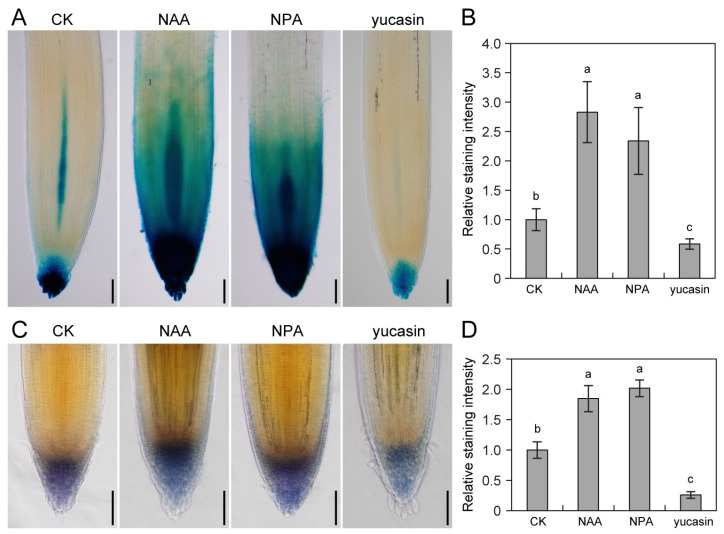
Auxin modulates starch granule accumulation in tomato roots. (**A**,**B**) DR5:GUS expression in tomato roots after 6 h treatment with 10 nM NAA, 1 μM NPA, or 50 μM yucasin, with DMSO as the CK. Analysis of DR5:GUS expression through GUS staining (**A**), and relative staining intensity (mean ± SE, n ≥ 8) measured using ImageJ software (**B**). (**C**,**D**) Starch granule accumulation in tomato roots after 6 h treatment with 10 nM NAA, 1 μM NPA, or 50 μM yucasin, with DMSO as the CK. Observation of starch granules through Lugol staining (**C**) and relative staining intensity (mean ± SE, n ≥ 8) measured using ImageJ software (**D**). Different letters indicate statistical differences (*p* < 0.05) as determined by Duncan’s multiple-range tests for one-way ANOVA. Bars, 100 μm.

The regulatory influence of auxin on starch granule accumulation has been elucidated in Arabidopsis roots, underscoring the crosstalk between auxin and starch dynamics [30]. Delving into the specific impact of auxin on starch granule formation and accumulation in tomato roots, the levels of auxin and starch were assessed using DR5:GUS and lugol stain, respectively. Comparative analysis revealed that the application of the auxin analog NAA significantly elevated auxin response signaling and promoted starch accumulation in root apical tissues compared to the CKs (Figure 5). Similarly, treatment with NPA robustly enhanced both auxin and starch granule accumulation (Figure 5), indicating that auxins play pivotal roles in fostering starch granules synthesis and buildup within root tip cells. Contrarily, the exogenous application of the auxin biosynthesis inhibitor yucasin markedly diminished both the auxin response and starch granule accumulation (Figure 5). These findings underscore the regulatory influence of auxin in shaping the perception of gravity signals through its modulation of starch granule accumulation in tomato root tip cells.

### 2.5. Transcriptome Analysis Revealed Insights into Tomato Root Gravitropism

**Figure 6:**
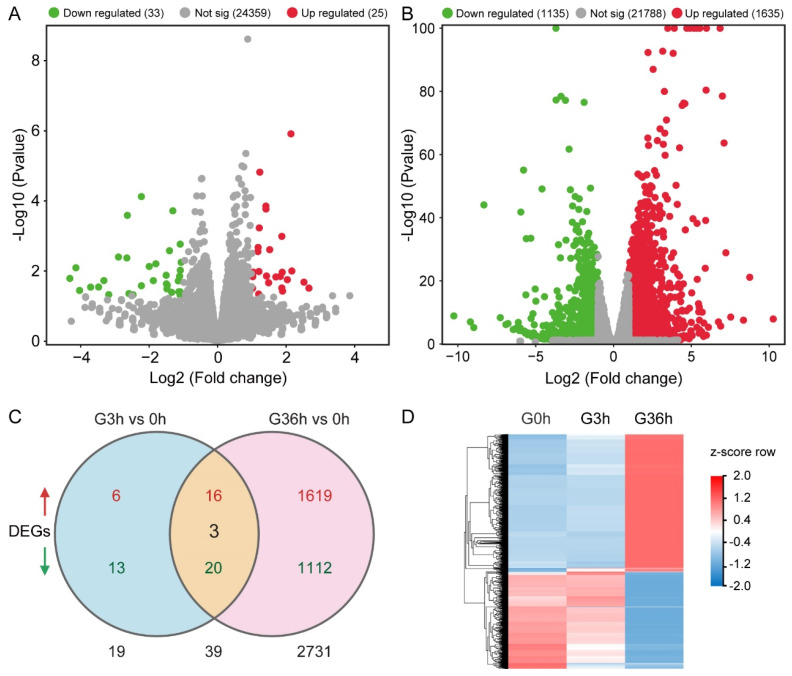
Transcriptomic analysis of the response to gravity stimulation. (**A**,**B**) RNA-Seq scatter plot displaying differentially transcribed genes (downregulated and upregulated genes) in tomato seedling roots after 3 h (**A**) or 36 h (**B**) of gravity stimulation. (**C**) In total, 2789 DEGs were identified from roots of tomato seedlings, including 58 and 2770 DEGs in roots exposed to gravistimulation for 3 and 36 h, respectively. Venn diagrams revealing the overlap (39 genes) between 3 and 36 h of gravistimulation-regulated genes. Among these 39 differential genes, 16 were upregulated, 20 were downregulated, and only 3 showed opposite expression patterns in the root between 3 and 36 h of gravistimulation. Red arrow signifies upregulated genes, and green arrow signifies downregulated genes. (**D**) Heatmap illustrating the 2789 DEGs. Three biological replicates. |Log2(Foldchange)| ≥ 1, *p*-value < 0.05.

Unraveling the intricacies of root gravitropism, we conducted RNA-seq on tomato roots subjected to gravity stimulation to elucidate the underlying regulatory mechanisms. The analysis of differentially expressed genes (DEGs) shed light on the transcriptional landscape in response to gravity stimulation. Upon 3 h of gravity stimulation, a modest 58 DEGs emerged, reflecting the subdued root gravitropic response in tomato (Figure 6A). Among these, 25 genes exhibited upregulation, while 33 displayed downregulation (Figure 6A). Extending the gravity exposure to 36 h resulted in a substantial shift, with 2770 DEGs identified—1635 genes upregulated and 1135 genes downregulated (Figure 6B). Venn diagram revealed that 2789 differentially expressed genes were involved in the root response to the gravistimulation (Figure 6C). Among them, 19 differentially expressed genes were detected only at 3 h of gravity stimulation, and as many as 2731 differentially expressed genes were detected at 36 h. Notably, a subset of 39 genes changed at both 3 and 36 h of gravity stimulation. Expression analysis unveiled that 36 genes exhibited similar expression patterns at both time points, with 16 upregulated and 20 downregulated genes. Only three genes displayed opposite expression patterns (Figure 6C). This suggests that these 39 genes are involved in sustained gravity responses.

A heat map analysis of 2789 DEGs revealed that the expression pattern of many DEGs changed significantly after 36 h compared with 3 h of gravity stimulation, suggesting a lagging molecular response (Figure 6D). This is consistent with the slow gravitropic response phenotype of tomato roots.

### 2.6. Starch and Sucrose Metabolism, Auxin Signaling, and Cell Wall Modification Are Important Pathways That Regulate the Response of Tomato Roots to Gravity Signals

**Figure 7:**
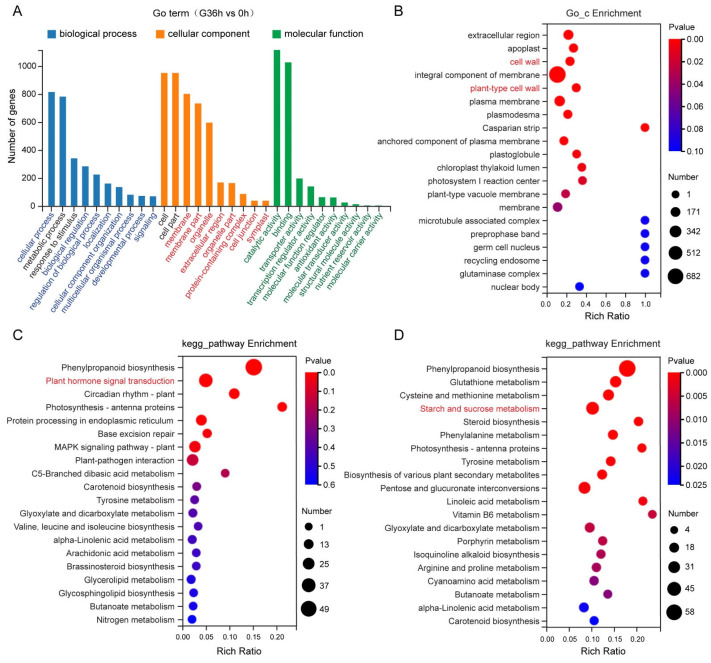
GO and KEGG analysis of DEGs in response to gravity stimulation. (**A**) GO enrichment results for DEGs in tomato seedling roots at 36 h after gravistimulation. (**B**) Enrichment analysis of DEGs in cellular components after 36 h of gravity stimulation. (**C**,**D**) KEGG enrichment analysis of DEGs in tomato seedling roots before and after gravity stimulation in response to stimulus (**C**) or metabolic process (**D**).

**Figure 8:**
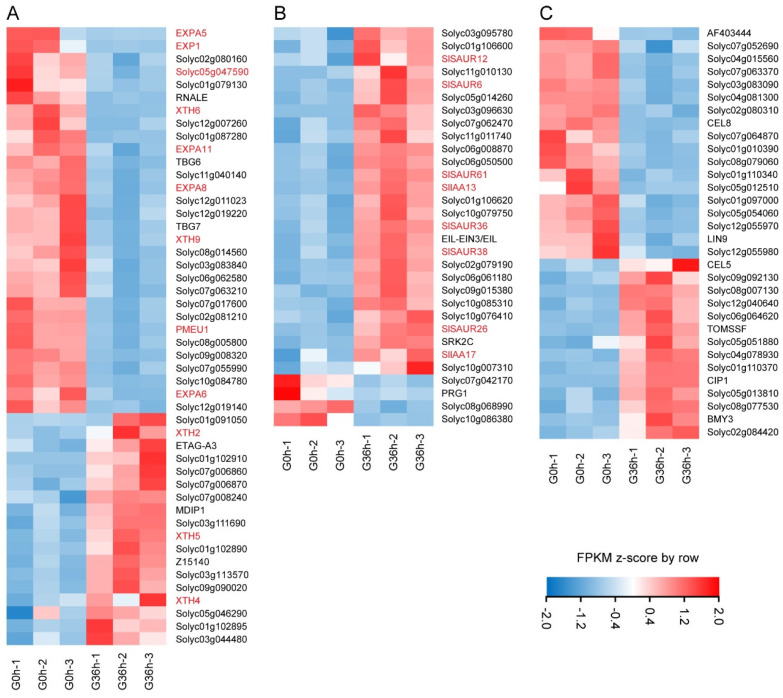
The heat map of DEGs related to cell wall modifications (**A**), plant hormone signal transduction pathways (**B**), and starch and sugar metabolism pathways (**C**).

Delving into the functional annotation of the DEGs through Gene Ontology analysis unveiled dominant subcategories in biological processes (BPs), encompassing cellular and metabolic processes, responses to stimuli, biological regulation, regulation of biological processes, localization, and cellular component organization (Figure 7A). The terms “cellular process, metabolic process, and response to stimulus” were significantly enriched in gravistimulated roots (Figure 7A). The DEGs included in the term “response to stimulation” are mainly enriched in the phenylpropanoid biosynthesis and plant hormone signal transduction pathways (Figure 7C). Analysis of the DEGs in the plant hormone signaling pathway revealed that the key genes responding to auxin are involved. Notably, the expressions of *SlSAUR6*, *SlSAUR12*, *SlSAUR26*, *SlSAUR36*, *SlSAUR38*, *SlSAUR61*, *SlIAA13*, and *SlIAA17* were all upregulated (Figure 8B). This suggests that auxin signaling acts as a positive response to gravistimulation. The DEGs included in the term “metabolic process” are mainly enriched in the phenylpropanoid biosynthesis, glutathione metabolism, cysteine and methionine metabolism, and starch and sucrose metabolism pathways (Figure 7D). Analysis of DEGs in the starch and sucrose metabolism pathways identified 32 DEGs involved in the root response to gravity signals (Figure 8C).

Cellular component (CC) terms highlighted the terms cell, cell part, membrane, organelle, extracellular region, organelle complex, and cell junction and symplast (Figure 7B). The term “cell wall” was significantly enriched in the samples. Specifically, there were 48 DEGs in the term “cell wall” in gravistimulated tomato roots (Figure 8A). Among them, 17 DEGs were upregulated, and 31 DEGs were downregulated (Figure 8A). The upregulated DEGs included *XTH2*, *XTH4*, and *XTH5*, and the downregulated DEGs contained *EXP1*, *EXPA5*, *EXPA6*, *EXPA8*, *EXPA11*, *XTH6*, *XTH9*, *PMEU1*, and *Solyc05g047590* (Solanum lycopersicum Pectinesterase) (Figure 8A). These comprehensive snapshots suggest the involvement of auxin signaling, starch and sucrose metabolism, and cell wall modification in the intricate dance of root gravitropic responses.

To verify the reliability of RNA-Seq, 9 DGEs were randomly selected for qRT-PCR. The results indicate that the expression patterns of these candidate genes are consistent with RNA-seq (Figure 8 and Figure S3).

Our future endeavors will involve transcriptome analysis to unravel the regulatory intricacies governing tomato root gravitational growth. This exploration will unveil key transcription factors and unravel the auxin signal regulatory networks underpinning the plant’s intricate mechanism for perceiving and responding to gravity signals.

## 3. Discussion

Auxin plays a crucial regulatory role in root gravitropism [20,25,34,35]. Our exploration, involving the exogenous application of auxin or analogs, aligns with previous findings in Arabidopsis mutants or transgenic plants exhibiting altered auxin levels, resulting in shorter root lengths and subdued gravitropic responses (Figure 4 and Figure S4) [25]. Differences in the effects of exogenous IAA and NAA on the gravitropic responses of tomato roots were observed. Specifically, NAA treatment appears to promote the gravitropic response initially and reduces root gravitropic behavior with prolonged treatment (Figure 4B). In contrast, IAA consistently exerted an inhibitory effect throughout the observation period (Figure 4A). This discrepancy may be attributed to the distinct mechanisms by which NAA and IAA enter cells. NAA crosses the plasma membrane via diffusion, whereas IAA relies on auxin influx carriers for cellular uptake [23]. However, further research is necessary to verify this hypothesis. Another explanation for this phenomenon may be attributed to the auxin-induced starch granule accumulation, enhancing its sensitivity towards gravitational signals (Figure 5). During the subsequent gravitropic response, there was a significant attenuation in the bending ability of tomato roots, indicating that auxin played a pivotal role at this stage by inhibiting gravitropic curvature. Furthermore, the tomato seedling roots treated with NAA were significantly shorter than those in the untreated group (Figure S4). This suggests that NAA’s behavior of reducing root gravity is related to its inhibition of root growth during the later stage.

The mutant *yucQ*, associated with the auxin biosynthesis pathway, further substantiates the critical roles of auxin in root development and gravitropism, displaying severe defects in both aspects, including abnormal gravity growth and short root phenotypes [25]. Notably, the *yuc* single mutant, in contrast, showcased no overt gravity growth defects [25]. Our present study mirrors these insights, where the application of the auxin biosynthesis inhibitor kyn induced a delayed response in tomato roots to gravity in the early stage. Despite this delay, the tomato roots eventually completed the correct response to gravitational stimulus in the later stage (Figure 4C). Contrastingly, the auxin biosynthesis inhibitor yucasin treatment resulted in a prolonged and ultimately incorrect response to gravity stimulation (Figure 4D). These findings underscore the nuanced interplay of auxin levels, emphasizing their criticality in normal root growth and an accurate gravitropic response.

In contrast to the nuanced root gravitropic responses observed under exogenous auxin or auxin biosynthesis inhibitor treatments, impeding polar auxin transport emerges as a potent disruptor of root gravitropism. The auxin synthesized in the aboveground tissues is transported unidirectionally through the vascular system to the root tip. AUX1 and PIN2 facilitate basipetal auxin transport from the columella to the elongation zone [18,21]. NPA disrupts PAT by binding to PINs [36,37]. Treatments with NPA and TIBA induced aberrant responses in root gravitropism, showcasing the critical role of polar auxin transport in this phenomenon (Figure 4E,F and Figure S2). Our earlier findings, supported by the study of polar auxin transport-related mutants *auxl* and *pin2*, underscored the agravitropic phenotype in response to disruptions in PAT [31]. Our forthcoming investigations will delve deeper into understanding the contribution of SlLAX1 and SlPIN2 to root gravitropism in tomatoes, elucidating the specific regulatory mechanisms involved. These findings underscore the profound impact of disrupting the polar transport and distribution of auxins, emphasizing their paramount importance in governing the intricate dance of root gravitropism.

The orchestration of the auxin concentration gradient in response to gravistimulation unfolds with discernible temporal nuances, distinguishing the root dynamics of tomato and Arabidopsis (Figure 2 and Figure 3; [38]). Previous findings underscored the swift establishment of evident polar auxin distribution in Arabidopsis root tips merely 3 h post-gravity stimulation [31]. In stark contrast, tomato roots manifested a conspicuous polar auxin distribution after 12 h of gravity stimulation (Figure 3). The results are consistent with the phenotype of a lagging response of tomato roots to gravity (Figure 2). Transcriptome analysis illuminated this temporal dichotomy, revealing 58 DEGs after 3 h of gravity stimulation in tomato roots (Figure 6). However, this number dramatically surged to 2770 DEGs after 36 h of gravity stimulation (Figure 6). As expected, our results revealed that the abundance of auxin-responsive genes (*SAURs*, *IAAs*) changed significantly after 36 h of gravity stimulation (Figure 8B and Figure S3). This stark contrast in transcriptomic responses elucidates the significantly slower gravitropic reaction exhibited by tomato roots. The duration required for auxin to establish a polar distribution in tomato roots (Figure 3A) is longer than that observed in Arabidopsis [31]. Furthermore, the delayed response of DEGs to gravity stimulation may contribute to the slower gravitropic response of tomato roots compared to Arabidopsis. In addition, the pronounced disparities in root morphology between tomato and Arabidopsis constitute a critical factor contributing to the divergent root gravitropic responses. Specifically, different numbers of cortex layers are present in the primary roots of tomato and Arabidopsis. Tomato roots exhibit a greater number of cortical cell layers compared to Arabidopsis [9,39,40]. Additionally, tomato roots possess an exodermis, which is lacking in Arabidopsis [40]. The tomato root has more cortical cell layers than that of Arabidopsis, which could be the reason for a slower response to changes in position relative to the gravity vector, as more cell layers need to adapt to growth/expansion. Starch granules nestled within root columella cells emerge as pivotal signaling entities, orchestrating the intricate dance of plant responses to gravity signals [11,13]. The regulatory role of auxins in the gravitropic response extends to its influence on starch synthesis and accumulation. In Arabidopsis, pre-treatment with the NAA or NPA heightened the auxin response and starch synthesis, bolstering root gravitropic reactions [30]. Conversely, the application of the auxin biosynthesis inhibitor kyn dampened the auxin response and starch synthesis, resulting in reduced root gravitropism [30]. Mirroring Arabidopsis outcomes [30], our investigation into the effects of exogenous NAA, NPA, TIBA, or yucasin treatment on the auxin response and starch granule accumulation in tomatoes yielded parallel results (Figure 5 and Figure S5). However, the response of tomato roots to these treatments showcased intriguing divergences. NAA or yucasin treatment curtailed the root gravitropic response, while NPA treatment induced pronounced defects in root gravitropism (Figure 4). The observed phenotype in NPA-treated tomato plants aligned with the Arabidopsis *pin2* mutant, characterized by excessive auxin accumulation and starch granules in the roots alongside gravitropic response defects (Figure 5; [21]). The varied experimental treatments and materials may explain the seemingly contradictory outcomes. Short-term NAA or NPA pre-treatment in Arabidopsis bolstered the gravitropic response [30], contrasting with our findings, where long-term NAA treatment weakened the root gravitropic response, and extended NPA treatment mimicked the severe gravitropic defect observed in the *pin2* mutant rather than eliciting a rapid response (Figure 4; [21]). This variance underscores the nuanced interplay of hormones and treatment durations in shaping root gravitropic dynamics, adding complexity to our understanding of this intricate regulatory network. Our results suggest that auxin mediates gravity signals through feedback regulation of starch accumulation, ultimately regulating root gravitropic growth. Auxin enhances starch synthesis and accumulation in columella cells by upregulating the expression of starch synthase genes [30]. Research has demonstrated that starch deposition directs the relocalization of LAZY to the bottom of columella cells [15]. LAZY modulates the gravitropic response by regulating PIN-mediated lateral auxin transport and asymmetric auxin distribution [12,16,41]. However, the precise mechanism by which LAZY mediates PAT remains to be elucidated.

Transcriptome analysis revealed that many DEGs related to cell wall modifications were involved in the gravitropic response of roots. In particular, several cell wall expansion proteins were identified, all downregulated during the gravitropic response of roots, including *EXP1*, *EXPA5*, *EXPA6*, *EXPA8*, and *EXPA11* (Figure 8A and Figure S3). The cell wall controls cell growth, and expansion regulates cell growth by controlling cell wall extension [42]. This suggests that cell expansion is inhibited in gravistimulated roots. In plants, the auxin signaling pathway mediated by TIR1/AFBs-AUX/IAAs-ARFs has been demonstrated to activate the expression of SAURs to regulate cell wall loosening and cell growth [24,43,44]. It was shown that the expression of *SlIAAs* and *SlSAURs* was significantly upregulated in gravistimulated roots (Figure 8B and Figure S3), indicating the enhancement of auxin signaling. These results imply an auxin–cell wall-mediated signaling pathway for the gravitropic response of roots. Despite the identification of numerous genes responsive to gravity stimulation, the mechanisms by which cells on the lower and upper sides of the root respond to gravity signals differ significantly. Specifically, cell elongation on the lower side of the root is inhibited, while the growth of cells on the upper side remains unaffected. To elucidate this precise regulatory mechanism, the detection of genes that are asymmetrically expressed in cells on the upper and lower sides of the root will be a focus of attention.

## 4. Materials and Methods

### 4.1. Plant Material

Tomato lines (*Solanum lycopersicum* cv. M82, Alisa Craig (AC), Micro-Tom, and var. cerasiforme Aisheng) were utilized in this study. Micro-Tom expressing *DR5:GUS* was obtained from the *DR5:GUS* transgenic line (“VF36” background) [45] through four backcrosses with Micro-Tom. The wild-type Arabidopsis belonged to the Columbia (Col-0) ecotype. Tomato and Arabidopsis seeds underwent sterilization with 1% NaClO for 10 min, followed by rinsing with ddH_2_O five times. They were then sown on solid medium containing 1/2MS salts, 1% sucrose, and 0.8% agar for germination in the darkness and cultured vertically. After germination, the seedlings were grown vertically in a plant climate incubator with a 14 h light/10 h dark (25 °C) cycle at 70% humidity.

### 4.2. Root Gravitropism Assays

Four-day-old tomato or Arabidopsis seedlings were transplanted to 1/2MS solid medium, vertically cultured for 12 h, and then underwent a 90° rotation for gravity stimulation. To eliminate the impact of light on plant tropism, the seedlings were grown in darkness. The bending angles of the root tips were documented by scanning the seedlings at specified intervals. The bending angle was calculated using ImageJ software (https://imagej.net/ij).

### 4.3. Hormone or Inhibitor Treatments

For chemical treatments, tomato seedlings were transferred to fresh 1/2MS with various chemicals. To maintain the consistency of vertical orientation and prevent variations in position caused by seedling movement, the seedlings were initially vertically cultured on a solid medium for 12 h and then rotated 90° for gravity stimulation. Bending angles were recorded by scanning the plates and capturing images at designated time points each day. Ethanol was used to dissolve IAA, and dimethyl sulfoxide (DMSO) was employed for dissolving 1-naphthaleneacetic acid (NAA), L-kynurenine (kyn), 5-(4-chlorophenyl)-4H-1,2,4-triazole-3-thiol (yucasin), 1-N-naphthylphthalamic acid (NPA), and 2,3,5-triiodobenzoic acid (TIBA). Final concentrations were 1, 5, 10, and 20 nM for IAA and NAA; 0.5, 1, 2, and 4 μM for kyn; 10, 20, 50, and 100 μM for yucasin; 0.1, 0.5, 1, and 5 μM for NPA; and 0.1, 0.5, 1, and 2 μM for TIBA.

### 4.4. GUS and Lugol’s Staining

Four-day-old seedlings of Aisheng or DR5:GUS transgenic tomatoes underwent pretreatment in a liquid 1/2 MS solution containing NAA (10 nM), yucasin (50 μM), and NPA (1 μM) for 6 h before being subjected to GUS or Lugol’s staining. For GUS staining [31], tomato seedlings were immersed in GUS staining solution at 37 °C for 14 h. The samples were fixed in 75% ethanol/25% acetic acid for 3 h. Lugol’s staining followed previously described methods [30]. Four-day-old Aisheng tomato seedlings were submerged in Lugol’s staining solution (w/w/v; I_2_:KI:H_2_O = 1:2:20) for 3 min and then washed with ddH_2_O.

Subsequently, tomato seedlings were transferred to a clearing solution (chloral hydrate/ddH_2_O/glycerol = 8:3:1) and photographed using a Zeiss microscope (Axio Imager.A2, Germany).

### 4.5. RNA-Seq Analysis

For transcriptome analysis, tomato seedlings were subjected to a 90° rotation for either 3 or 36 h of gravity stimulation. The roots were excised from the seedlings, snap-frozen in liquid nitrogen, and kept at −80 °C. Samples underwent sequencing and analysis using the BGISEQ platform. Total RNA was extracted using a Plant Total RNA Purification Kit (TIANGEN, Beijing, China). The reference genome version used was ITAG 4.0 (https://solgenomics.net). Each indicated time point was independently repeated three times.

## 5. Conclusions

**Figure 9:**
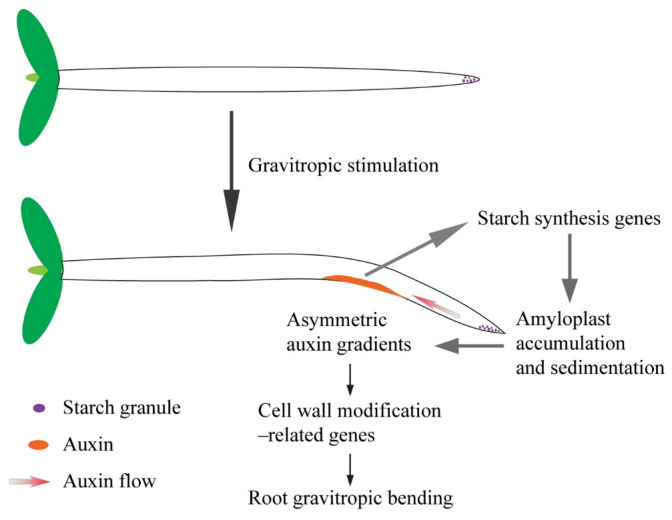
Schematic diagram of the starch granule–auxin-mediated root gravitropic response. When the plant is stimulated by gravity, the starch granules located in the columella of the root settle to the new bottom of the cells due to the relatively high proportion, stimulating lateral polar auxin transport mediated by AUX1 and PIN2 [31]. Excess auxin inhibits the elongation of cells in the elongation zone by regulating the expression of cell wall-related genes. In addition, auxin can affect the response of the root to gravity signals by feedback regulation of the synthesis and accumulation of starch granules in the root tip [30].

Based on the above results and previous research reports, a signal regulatory pathway for plant gravitropic growth was proposed (Figure 9). Briefly, plants sense gravity signals through the sedimentation of starch granules, which triggers the lateral transport of auxin from the root tip to the EZ. Auxin inhibits the elongation of cells at the lower side of the EZ by regulating the expression of genes associated with cell wall modification, thereby driving the gravitropic bending of roots. The anchoring effect of gravitational root growth not only ensures plant stability but also reduces soil erosion and enhances water and fertilizer use efficiency, thereby playing a crucial role in promoting high-quality agricultural yields and combating climate change. This exploration aims to unveil key genes and unravel the auxin signal regulatory networks that underpin the plant’s intricate mechanism for perceiving and responding to gravity signals.

## Data Availability

Data are contained within the article.

## Supplementary Materials

The following supporting information can be downloaded at https://www.mdpi.com/article/10.3390/plants14071020/s1, Figure S1. Root gravitropic bending rates of tomatoes. Figure S2. Effect of NAA, NPA, and yucasin on auxin responses during root gravitropic response. Figure S3. The expression of 9 DEGs identified by RNA-seq was verified by qRT-PCR. Figure S4. Impact of IAA or NAA on root gravitropic response of tomato. Figure S5. TIBA affects the accumulation of starch granules in tomato roots. Table S1. Primers used in this study.
