# Supplementary material for: Auxin Controls Root Gravitropic Response by Affecting Starch Granule Accumulation and Cell Wall Modification in Tomato
Source: Plants (Basel). 2025 Mar 25;14(7):1020. doi: 10.3390/plants14071020 (PMC11990612; doi:10.3390/plants14071020)
Supplement: Supplementary file 1 [file plants-14-01020-s001.zip › plants-3515650-supplementary.pdf]

Supporting information

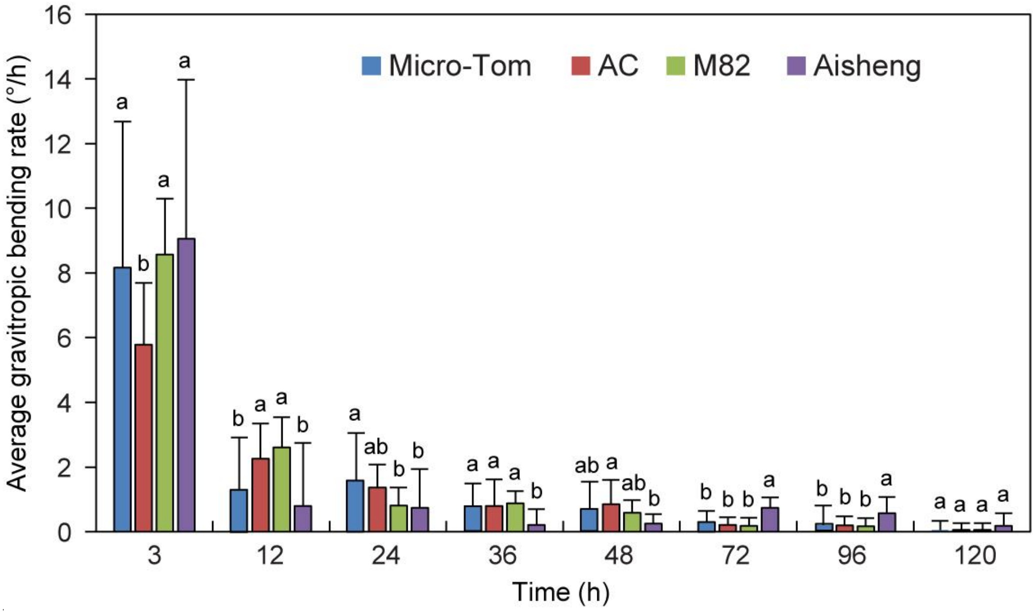

Figure S1. **Root gravitropic bending rates of tomatoes**

Four-day-old seedlings of the four tomato cultivars (Micro-Tom, AC, M82, and Aisheng) were rotated 90° for gravity stimulation, and average gravitropic bending rates were calculated at the indicated time points. Values represent mean ± SD of three biological replicates (6–8 seedlings per replicate). Different letters indicated statistically different ($p < 0.05$), Duncan's multiple range tests for one-way ANOVA.

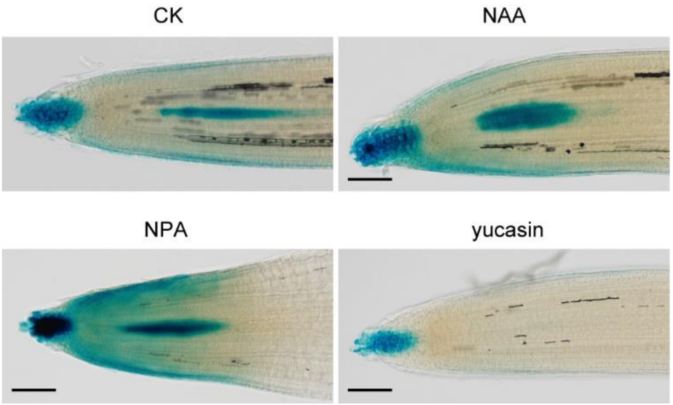

Figure S2. Effect of NAA, NPA and yucasin on auxin responses during root gravitropic response.

DR5:GUS-expressing tomato seedlings (4 days old) were transplanted to 1/2MS solid medium with 10 nM NAA (B), 1 μM NPA (C), or 50 μM yucasin (D), vertically cultured for 3 h, and then underwent a 90° rotation for gravity stimulation for 12 h. Bars, 200 μm.

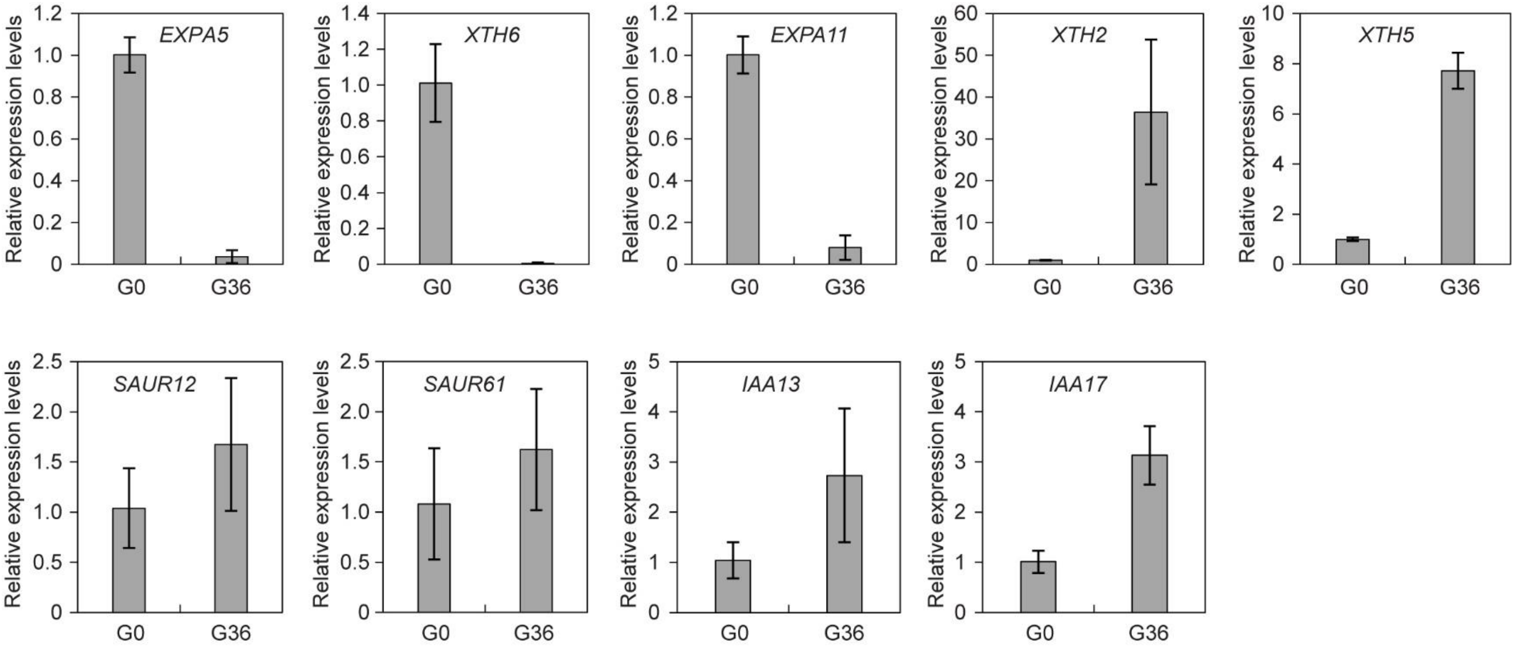

Figure S3. The expression of 9 DEGs identified by RNA-seq were verified by qRT-PCR.

Four-day-old tomato seedlings (Aisheng) were subjected or not (control, G0) to a 90° rotation for 36 h of gravity stimulation (G36). RNA was isolated from roots and used for detecting gene expression. qPCR analyses of the genes (DEGs in Figure 8) to validate these transcriptome sequencing data. The UBI3 gene was used as an internal control.

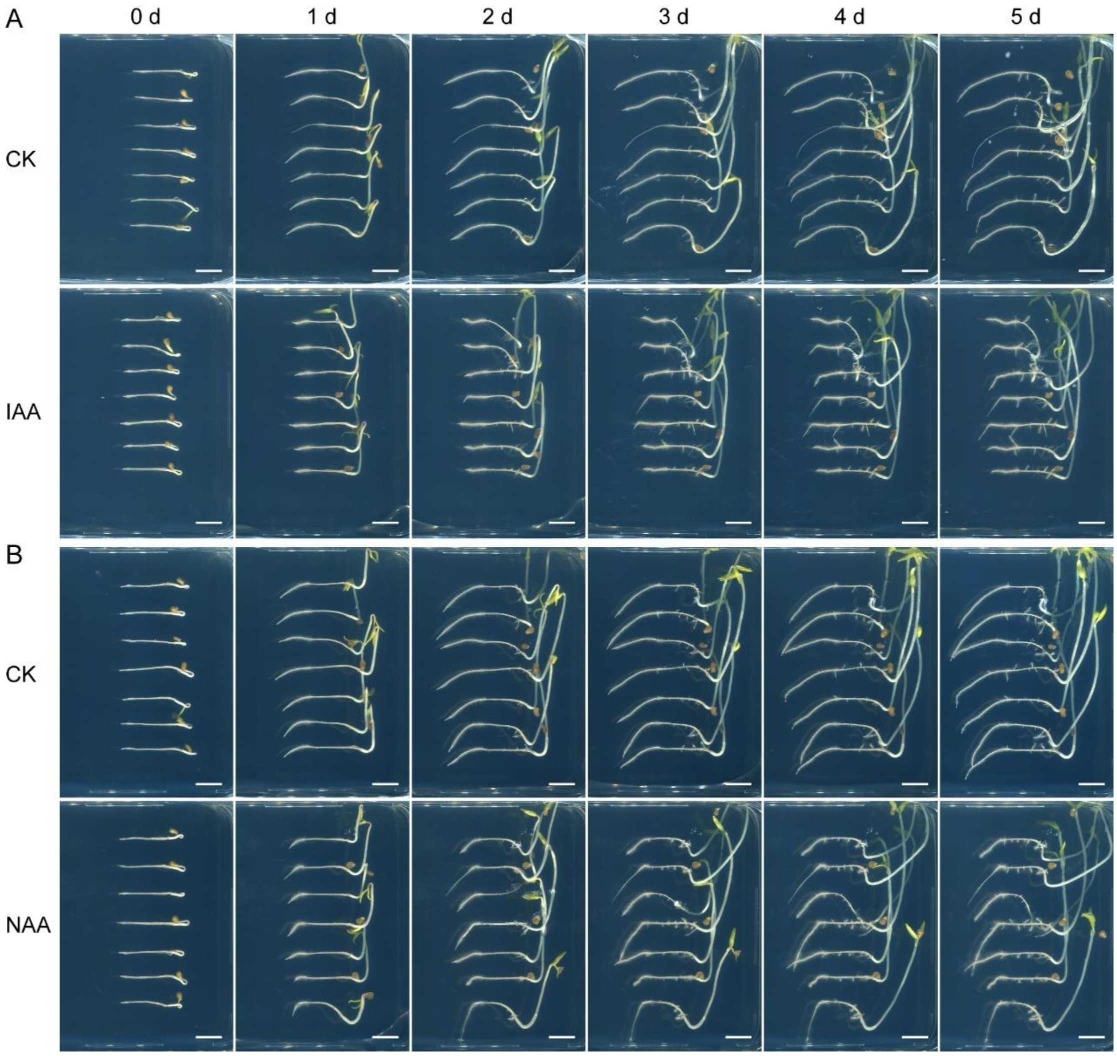

Figure S4. **Impact of IAA or NAA on root gravitropic response of tomato**

A, B. Four-day-old tomato (Aisheng) seedlings were transferred to solid medium containing 20 nM IAA (A) or NAA (B), cultured vertically for 12 h, and then the plates were rotated 90° for gravity stimulation. Seedlings were scanned at the indicated times. Scale bar = 1 cm.

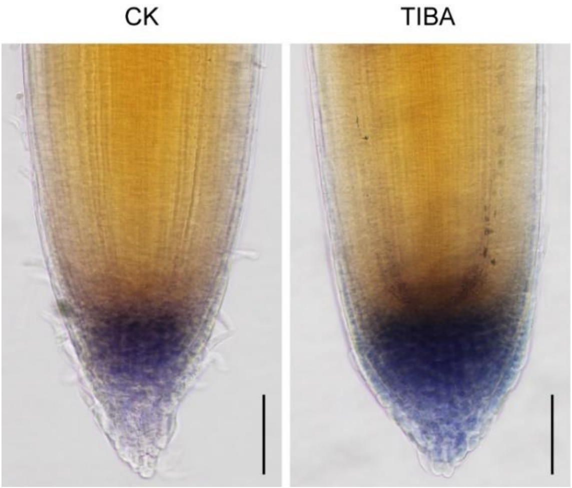

Figure S5. **TIBA affects the accumulation of starch granules in tomato roots**

Starch accumulation in the root tip of tomato (Aisheng) after 6 h treatment with 1 μM TIBA, with DMSO as the CK. Scale bar = 100 μm.

Table S1. Primers used in this study

Primer name	Sequence (5' to 3')
EXPA5_qF	CCTCTATTTATTCTCCACGATTGCT
EXPA5_qR	TGTTCGTTCCGTATCCCGTG
EXPA11_qF	TCATCTACTTTTCTTGGCTTAGACC
EXPA11_qR	TTCCCATAGCCACAAGCACC
XTH2_qF	ATGGGGTTATGGGAGGGTGA
XTH2_qR	TGCCAGCAGAATTACCAGGG
XTH5_qF	GGGATGCTTCAGATTGGGCT
XTH5_qR	AATCTCGCACTCTGGTGGTG
XTH6_qF	CATCTTGGGCAACGGAGGAA
XTH6_qR	TGCACCCACAACATAGCCTC
SAUR12_qF	CCCAAGGGTCATTTTGCTGTG
SAUR12_qR	CCATTGGATGGTCGAAGCCA
SAUR61_qF	GCACCGCAAGGTTGTTTTTG
SAUR61_qR	GAGCCATAACCCTTGCCCAT
IAA13_qF	GGAGTTGGCGGGAACGATAA
IAA13_qR	GCTTGTTCCTTTTCACCGCT
IAA17_qF	CAAGAATTATTTGATGCCTTAACCAA
IAA17_qR	ACTATTCAAAAGGTCCATCAGTTTCC
SlUBQ_qF	CACCAAGCCAAAGAAGATCA
SlUBQ_qR	TCAGCATTAGGGCACTCCTT
